# Synthesis of Benzofuran-2-One Derivatives and Evaluation of Their Antioxidant Capacity by Comparing DPPH Assay and Cyclic Voltammetry

**DOI:** 10.3390/molecules23040710

**Published:** 2018-03-21

**Authors:** Martina Miceli, Elia Roma, Paolo Rosa, Marta Feroci, M. Antonietta Loreto, Daniela Tofani, Tecla Gasperi

**Affiliations:** 1 Dipartimento di Scienze, Sezione di Nanoscienze e Nanotecnologie, Università degli Studi di Roma Tre, via della Vasca Navale 79, I-00146 Roma, Italy; elia.roma@uniroma3.it (E.R.); daniela.tofani@uniroma3.it (D.T.); 2 Dipartimento di Scienze e Biotecnologie Medico-Chirurgiche, Sapienza Università di Roma—Polo Pontino, Corso della Repubblica 79, 04100 Latina, Italy; p.rosa@uniroma1.it; 3 Dipartimento di Scienze di Base e Applicate per l’Ingegneria, Sapienza Università di Roma, via Castro Laurenziano 7, I-00161 Roma, Italy; marta.feroci@uniroma1.it; 4 Dipartimento di Chimica, Sapienza Università di Roma, p.le A. Moro 5, I-00185 Roma, Italy; mariaantonietta.loreto@uniroma3.it

**Keywords:** antioxidant activity, cyclic voltammetry, DPPH, domino reaction, benzofuran-2-ones

## Abstract

The present work aimed to synthesise promising antioxidant compounds as a valuable alternative to the currently expensive and easily degradable molecules that are employed as stabilizers in industrial preparation. Taking into account our experience concerning domino Friedel-Crafts/lactonization reactions, we successfully improved and extended the previously reported methodology toward the synthesis of 3,3-disubstituted-3*H*-benzofuran-2-one derivatives **9**–**20** starting from polyphenols **1**–**6** as substrates and either diethylketomalonate (**7**) or 3,3,3-trifluoromethyl pyruvate (**8**) as electrophilic counterpart. The antioxidant capacity of the most stable compounds (**9**–**11** and **15**–**20**) was evaluated by both DPPH assay and Cyclic Voltammetry analyses performed in alcoholic media (methanol) as well as in aprotic solvent (acetonitrile). By comparing the recorded experimental data, a remarkable activity can be attributed to few of the tested lactones.

## 1. Introduction

**Figure 1:**
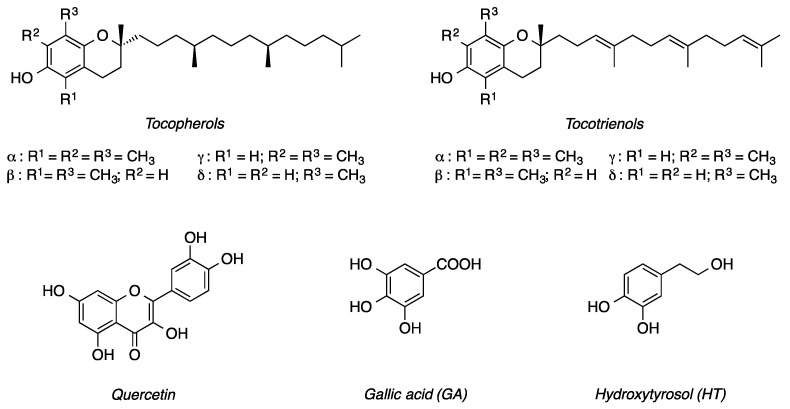
Examples of natural phenolic antioxidants.

The interplay between free radicals and antioxidants represents a crucial point in the clinical and nutritional research field [1,2,3,4,5]. Free radicals are responsible for oxidative stress, which is balanced by endogenous antioxidant defense mechanisms as well as by the ingestion of exogenous antioxidants [6,7]. In the human body, the overproduction of radical species can cause oxidative damage to fundamental biomolecules (i.e., DNA, lipids, proteins, carbohydrates, etc.), favoring cell apoptosis [8] and becoming a primary cause of several both chronic and degenerative diseases (i.e., aging related pathogenesis, neurological and cardiovascular diseases, skin disorders, cancer etc.) [9,10,11]. In order to prevent the undesirable outbreaks of such issues, various natural or synthetic antioxidants are nowadays used as dietary supplements (Smart Food), and are also considered candidate drugs for the reduction of the oxidative damage [12,13]. Among others, tocopherols and tocotrienols, different forms of Vitamin E [14], flavonoids (quercetin) [15,16], hydroxytyrosol (HT) [17], gallic acid [18,19], and their derivatives are noteworthy phenolic antioxidants (Figure 1) which have even shown fascinating antithrombotic activities (i.e., inhibition of LDL oxidation, platelet aggregation, and endothelial cell activation) [20]. Such outstanding actions probably rely on the catechol system, which is a common feature in the above bioactive compounds [21]. Unfortunately, the currently employed products are seldomly used not only as dietary supplements but also as stabilizers in foods, cosmetics, or industrial preparations, mainly due to their frequent degradation. Consequently, the research of more stable antioxidants with a broad application scope remains a challenging endeavor of great interest.

Prompted by these considerations, we planned the synthesis of new generation antioxidant compounds and the evaluation of their antioxidant activity through a deep comparison between DPPH assay and Cyclic Voltammetry analyses. Specifically, we design potential antioxidant molecules characterized by the 3,3-disubstituted-3*H*-benzofuran-2-one framework decorated with one or more hydroxyl groups on the aromatic ring. Such a distinguishing scaffold not only is found in key intermediates towards the synthesis of biologically active molecules [22,23], but occurs also as a common feature of many natural medicinal products, among which the antioxidant species play a critical role [24,25,26,27]. Indeed, the 3*H*-benzofuran-2-ones, or 2-coumaranones, are a significant class of heterocyclic molecules highly widespread in nature, consisting of a benzene fused with a furan-2-one ring [27,28,29,30]. For example, the 3,3-disubstitued 2-coumaronone scaffold is a prominent structural motif in many natural compounds, such as in yuccaol A–E, isolated from *Yucca Schidigera*, which have exhibited antioxidant, radical scavenging, and inflammatory properties, as well as inducible NO synthase (iNOS) expression and platelet aggregation inhibition [31,32,33,34,35].

## 2. Results and Discussion

### 2.1. Synthesis

Within this context, although several elegant strategies for the construction of the 3*H*-benzofuran-2-one scaffold [23,36,37,38] have been reported, the number of approaches to the corresponding 3-hydroxy derivatives is much more limited. Nevertheless, following our recent development of alternative, short, and practical route to 3-hydroxy-3*H*-benzofuran-2-one [39,40], we have figured out the possibility of obtaining various phenolic derivatives by domino reaction involving a first Friedel–Crafts alkylation and a subsequent intramolecular lactonization. Specifically, the reaction was performed employing the polyphenols **1–6** as substrates and either ketomalonate **7** or 3,3,3-trifluoromethyl pyruvate **8** as the electrophilic counterpart in the presence of TiCl_4_ (10 mol%) as catalyst, which should activate the alkylating agent as well as the postulated intermediate **A** (Table 1).

As depicted in Table 1, employing the previously optimized conditions, only the polyphenols **1–3** furnished the desired bicyclic compounds **9**–**11** with good to high yields (up to 81%, entries 1–3), when the ketomalonate **7** was used as alkylating agent; meanwhile substrates **4**–**5**, owing the second hydroxyl group in the *ortho* position (**4**) or two OH substituents (**5** and **6**), led to a complex reaction mixture where the corresponding products **12** and **13**, initially detected at the ^1^H-NMR, were isolated in very poor yields, mainly due to their instability (entry 4–6). Additionally, the more reactive 3,3,3-trifluoromethyl pyruvate **8** provided exclusively the benzofuran-2-one **17** in acceptable amount (61%, entry 9). On the contrary, the same electrophile **8** reacted slowly with hydroxyl phenols **2** and **3**, which furnished the 3-hydroxy lactones **15** (entry 7) and **16** (entry 8) in moderate yields (35% and 28% respectively) and almost no conversion was detected starting from substrates **4**–**6** (entries 10–12).

In light of these outcomes, we supposed that the installation of a strong interaction between the employed Lewis acid (TiCl_4_) and the several hydroxyl groups bounded to the substrates should establish various stable complexes by prohibiting any possible nucleophilic attack of the alkylating agent on aromatic ring. For this reason, we carried out additional investigations in order to optimize the domino Friedel–Crafts/lactonization reaction between polyphenols **1**–**6** and 3,3,3-trifluoromethyl pyruvate **8** as alkylating agent. Specifically, we employed *p*-hydroxyphenol (hydroquinone) **1** as the model substrate and different reaction conditions. The observed results are summarized in Table 2.

Firstly, we examined catalytic amounts of two different Lewis acids, precisely, BF_3_·Et_2_O (entries 1 and 2) and AlCl_3_ (entry 3), but both of them were neither effective nor efficient, leading to a complex reaction mixture without any trace of the target compound. Subsequently, benzoic acid (entries 4 and 5) and camphorsulfonic acid (CSA) (entries 6 and 7), were tested, varying both their amounts and the reaction temperature, but unfortunately the starting materials were recovered almost quantitatively. A similar result was achieved using a catalytic amount of either DBU (entry 8) or acetic acid in dichloromethane, where they did not catalyse any reaction and even after 72 h the starting material was recovered.

The best results were observed by exploiting the approach previously reported by Dyachenko et al. [41,42], who employed acetic acid as solvent. With our delight, benzofuran-2-one **15** was obtained with a 45% yield keeping the temperature at 120 °C for 12 h (entry 10). Differently to Lewis acids, the employment of acetic acid does not suffer from the formation of an unreactive substrate/catalyst complex. Therefore, the mechanistic pathway should involve (see Scheme SM-1): (i) the initial activation of the alkylating agent **8** by the acidic solvent that promotes (ii) the Friedel–Crafts alkylation at the *ortho* position of substrate **1** to obtain the key intermediate **A**; (iii) a subsequent acid-assisted intramolecular transesterification to deliver the expected 3-hydroxylactone **15**.

The subsequent optimization of the applied protocol particularly benefited from a more concentrated reaction mixture and decrease in the reaction time (entries 11 and 12). Finally, the desired lactone **15** was isolated in 80% yield after only 4 h at 120 °C and using 3 mL of acetic acid as solvent. In almost all cases, the desired product was successfully formed in quite good yield (up to 86%). Noteworthy, in phenol **3** containing a hydroxyl group in the *meta* position, the introduction of an alkyl group in the *ortho* position considerably enhanced the overall reactivity (86% yield, entry 14), with respect to the corresponding non-methylated phenol **2**, which gave the desired lactone **16** with 72% yield (entry 13), as well as the phenol **5**, which possesses two additional hydroxyl groups in both the *meta* positions and reacted even less efficiently furnishing the lactone **19** in 66% yield (entry 16). Moreover, the presence of a catecholic group (entries 15 and 17) did not interfere with the reaction path, as proven by compounds **18** and **20**, that were obtained in comparable yields (respectively 78% and 76%) after 4 h.

Additionally, hydroxyl phenols **4**–**6** were tested using AcOH as solvent and diethylketomalonate **7** as alkylating agent at high temperature (120 °C), but unfortunately a complex reaction mixture was obtained and the corresponding products **12**–**14** were not detected, which confirmed the extreme lability of such compounds.

### 2.2. Antioxidant Capacity Evaluation

In the last decades, there has been an increasing interest in antioxidants, principally in those proposed to prevent the potential injurious effects of free radicals [43]. Therefore, it is very appealing to have convenient and quick methods for estimating the efficacy of a substance as antioxidant. For this purpose, numerous in vitro methods have been developed to evaluate different antioxidant effects [44,45]. Depending upon the reactions involved, these assays can be classified into assays based on hydrogen atom transfer (HAT) reactions, and assays based on electron transfer (ET). The former assays analyse competitive reaction kinetics, quantified through the kinetic curves, while the ET-based assays monitor one redox reaction with the oxidant as an indicator of the reaction endpoint. Both methods are able to measure the radical (or oxidant) scavenging capacity, instead of the preventive antioxidant capacity of a sample [46]. In light of such features, we evaluated the antioxidant capacity of our compounds by DPPH assay and Cyclic Voltammetry analyses.

#### 2.2.1. DPPH Assay

**Figure 2:**
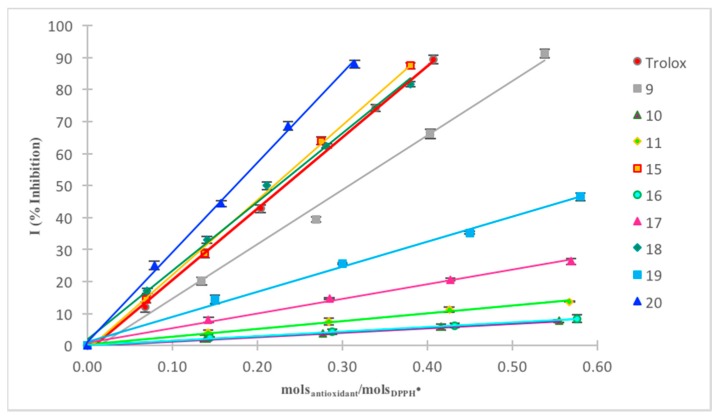
Regression line of the tested compounds **9**–**11** and **15**–**20** in DPPH assay in methanol.

The DPPH assaying of compounds **9**–**11** and **15**–**20** was performed using Trolox as reference compound. Actually, to achieve more reliable results and to obtain information on the antioxidant activity of these compounds, the assay was carried out both in alcoholic media (methanol, MeOH) as well as in an aprotic solvent (acetonitrile, ACN), i.e., solvents with different abilities in solvating molecules and forming hydrogen bonds. Specifically, to the degassed solutions (2 mL) of antioxidant, with a final concentration from 0 to 60 μM, were added 2 mL of 140 μM DPPH^•^ stock solution (70 μM DPPH final concentration). The solution was stirred at room temperature in the absence of light, and, after 60 min, the absorbance was measured at 517 nm. The working DPPH^•^ concentration was calculated from the absorbance response at 517 nm of the initial solution in the absence of antioxidant and using the calibration curves reported for methanol in Figure 1 and acetonitrile in Figure 2, respectively. The DPPH^•^ scavenging percentage or inhibition [I%] was calculated from the measured absorbance using the following expression:

$$I\left(\%\right)=\frac{\left(A-A_{0}\right)}{A_{0}}\;\times 100$$

where *A*_0_ and *A* represent respectively the absorbance in the absence of antioxidant and the absorbance at a given antioxidant concentration. The plot of *I*(%) versus the molar ratio of reagents (mols_antioxidant_/mols_DPPH•_) presented a linear response (ŷ = ax ± b) until the plateau was reached, that is, when the inhibition arrived to its maximum [44,47]. The predicted relative IC_50_ (rIC_50_, the half inhibitory concentration in terms of mols_antioxidant_/mols_DPPH•_) was estimated replacing the ŷ-value by 50 in the regression line (see the Supplementary Material). Sometimes it is more useful to discuss in terms of antiradical power (ARP), that is the inverse of rIC_50_, therefore the higher the ARP value is, the more efficient the antioxidant is.

##### DPPH Assay in Methanol

Figure 2 shows the plot of inhibition percentage versus moles antioxidant/moles DPPH^•^ for the synthesized compounds **9**–**11** and **15**–**20**.

By comparing the recorded absorbances (Figure 2 and Table 3), remarkably compounds **9**, **15**, **18**, and **20** achieve a ratio molar (mols_antioxidant_/mols_DPPH__•_) between 0.18 and 0.31, which is quite close to the value obtained with Trolox (0.23), whereas the other synthesised benzofuran-2-ones (**10**, **11**, **16**, **17**, and **19**) exhibit a poor to moderate reactivity towards the DPPH^•^. From the regression lines, the rIC_50_ in terms of mols_antioxidant_/mols_DPPH__•_ was subsequently estimated as well as the corresponding coefficient of determination R^2^, which undoubtedly validated the robustness of our analyses (Table 3). Additionally, the antiradical power (ARP), the stoichiometry, and the number of DPPH^•^ reduced were calculated, as reported in Table 3.

As depicted above, compounds **9**, **15**, **18**, and **20** presented rIC_50_ values from 0.18 to 0.31 (entries 2, 5, 8, and 10 respectively), representing the best antioxidants of the analysed series in the employed conditions. Specifically, compounds with a single phenolic group (i.e., **9**, **15**, and **18**) seemed to reduce approximately two molecules of DPPH^•^, whereas the benzolactone **20**, decorated with two hydroxyl groups on the aromatic ring, reduced almost three molecules of DPPH^•^. These experimental results highlight the possible involvement of the lactone ring in the reduction mechanism, although no exhaustive mechanistic study was undertaken. Conversely, the remaining compounds (i.e., **10**, **11**, **16**, **17**, and **19**), bearing a hydroxyl group in *meta* position (with respect the oxygen of the lactone ring), show quite high values of rIC50 (0.62–3.62) and consequently, a limited antioxidant capacity toward DPPH^•^ (entries 3, 4, 6, 7, and 9 respectively). In order to further validate these outcomes, the DPPH assay was also carried out in an aprotic solvent (acetonitrile), and the results were consequently compared to those obtained in alcoholic media.

##### DPPH Assay in Acetonitrile

**Figure 3:**
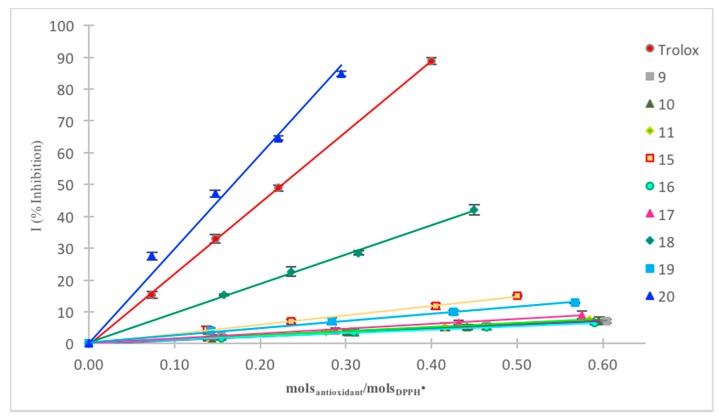
Regression line of the tested compounds **9**–**11** and **15**–**20** in DPPH assay in acetonitrile (ACN).

The assay was performed as described above, using acetonitrile as solvent (see experimental part for calibration curve of DPPH in ACN, SM). The absorbance measurements of each solution containing a known concentration of antioxidant and DPPH^•^ were collected. Figure 3 depicted the plot of inhibition percentage versus mols_antioxidant_/mols_DPPH__•_ for the synthesized compounds **9**–**11** and **15**–**20**.

By the comparison of achieved results (Figure 3 and Table 4), only compound **20** shows a ratio molar (mols_antioxidant_/mols_DPPH•_) of 0.17, which suggests an even better antioxidant capacity than that of Trolox, whereas the other synthesised benzofuran-2-ones (**9**–**11** and **15**–**19**) exhibit a lower reactivity towards the DPPH^•^. Table 4 shows the corresponding regression lines with the values of R^2^ as well as the rIC50 and all the deducible parameters, i.e., the ARP, the stoichiometric value, and the number of DPPH^•^ molecules that were reduced.

The results depicted above are surprisingly different from the previous ones recorded in methanol. Indeed, almost all the examined compounds showed an inadequate antioxidant capacity toward DPPH^•^ in acetonitrile, since compounds **9**–**17** and **19** exhibited rIC_50_ between 1.69 and 4.47, not reducing any molecules of DPPH^•^. Conversely, compound **18** exhibited a better antioxidant capacity, although not to the level of Trolox, with a rIC_50_ of 0.54 and a number of reduced molecules of DPPH^•^ amounting approximately to one. Exceptionally, with our delight, the 3-hydroxy benzofuran-2-one derivative **20**, bearing two hydroxyl groups in *ortho* and *meta* positions, presented a rIC_50_ of 0.17, which reduced three molecules of DPPH^•^, as observed before in methanol.

These experimental results highlight the different behavior of compounds in the DPPH assay with respect to the employed solvent. In this regard, the rate constants for hydrogen atom abstraction from compounds **9**, **15**, **18**, and **20** by the DPPH^•^ were determined both in methanol and acetonitrile. Such a kind of analyses should help us to verify if the obtained results could have been invalidated by solvent interference.

#### 2.2.2. Measurement of Rate Constants for the Reaction of Compounds **9**, **15**, **18**, and **20** with DPPH^•^

**Scheme 1:**
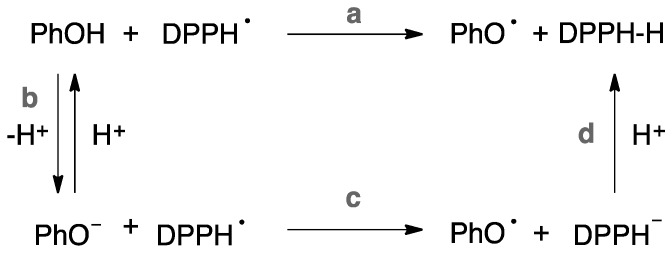
Reaction involved in the kinetic solvent effects.

Several studies on the kinetic solvent effects (KSEs) of DPPH^•^/phenol reactions established that the large KSEs observed for H-atom abstractions from phenols are mainly a consequence of hydrogen bonding to the solvent, when it is a hydrogen bond acceptor (HBA) [48,49,50]. Precisely, Ingold et al. [51,52,53] endorsed that the bimolecular rate constant enhancement (k^s^_PhOH/DPPH•_, Scheme 1, reaction a) is due to the partial ionization of the phenol in those solvents that supports an ionization process (Scheme 1, reaction b), especially alcohols, and a very fast electron transfer from the phenoxide anion to the DPPH^•^ (Scheme 1, reaction c), leading to profound kinetic consequences. Additionally, enhanced rate constants were a general feature of PhOH/DPPH^•^ reaction also for phenols with low p*K*_a_ values, in a non-hydroxylic, polar solvent, such as *n*-butyl ether, acetonitrile, THF, and DMSO.

In order to better comprehend the possible kinetic solvent effects in the performed DPPH assays of compounds **9**, **15**, **18**, and **20**, the rate constant measurement for the reactions of these compounds with DPPH^•^ was carried out. Practically, the decay of DPPH^•^ absorbance in the presence of a known concentration of phenols was followed at 517 nm and analysed as a pseudo-first-order process to yield the k_ex_/s^−1^. Because of this, the antioxidants were always used in large excess (final concentration 1–6 mM) over DPPH^•^ (final concentration 85 µM). Afterwards, the second-order rate constants (k^s^_PhOH/DPPH_^•^) of the synthesised compounds were evaluated by plotting the pseudo-first-order constant (k_ex_) versus antioxidant concentration: this plot is linear and its slope gives the bimolecular constants (see Supporting Material Table SM-5 and Table SM-6). Table 5 shows the k^s^ of analysed compounds, as well as the corresponding rIC_50_ are reported again for a more explicit comparison.

As shown above, compound **9** (entry 1) gave a high value of k^s^ in acetonitrile, although the corresponding rIC_50_ revealed a moderate antioxidant capacity. Moreover, even though the kinetic constant is lower in methanol than in acetonitrile, the significant value of k^s^ suggests a partial ionization of the phenol and slight interference by the employed solvent. Instead, compounds **15** and **18** (entries 2 and 3) display values of k^s^ quite in agreement with the corresponding rIC_50_: in methanol, a partial ionization of the phenols altered the results obtained with DPPH assay in methanol (rIC_50_ 0.22 and 0.24, respectively), while in acetonitrile, the value of rIC_50_ was reliable. Finally, the k^s^ values of compound **20** (entry 4), which presented a similar antioxidant capacity towards DPPH^•^ in both solvents, were mildly in disagreement, in fact in acetonitrile the k^s^ was higher than in methanol. Consequently, the obtained result in the DPPH assay, considering the reaction kinetics and possible solvent effects, were reassessed and benzofuran-2-one **20** presented a real quite good antioxidant capacity toward DPPH^•^.

#### 2.2.3. Cyclic Voltammetry

Cyclic voltammetry (CV) is an electrochemical technique frequently used (often along with DPPH assay) for the determination of the antioxidant activity of target compounds [54,55]. It consists of a potential scanning from a starting value to a final one, and then returning to the initial potential, while registering the current flowing between two electrodes. The potential at which an increase of current is registered corresponds to the oxidation (or reduction) of a species in solution. Thus, the oxidation potential measured by cyclic voltammetry can be taken as a measure of the ease of the oxidation process. Specifically, low oxidation potentials are associated with a greater facility of a given molecule for electro-donation and thus to act as antioxidant [54,56,57,58].

Moreover, CV can be carried out in aqueous medium as well as in organic solvents, e.g., in the presence of a supporting electrolytes to ensure the electrical conductivity of the solution [58].

The redox chemistry of the synthesized compounds **9**–**11** and **15**–**20** was evaluated using CV, recording the voltammograms in aqueous medium (H_2_O) as well as in an organic solvent (acetonitrile) and comparing the first oxidation peak potential to that of Trolox as a reference compound. Table 6 summarizes the observed results.

##### Cyclic Voltammetry in Aqueous Medium of Compounds **9**–**11** and **15**–**20**

The CV in aqueous medium was performed in a cell containing a three-electrode system: (i) the reference electrode (Saturated Calomel Electrode, SCE); (ii) the working electrode (Glassy Carbon, GC); and (iii) a counter electrode (platinum wire). Each antioxidant (2 × 10^−3^ M final concentration), dissolved in EtOH, was added to 40 mL of a solution of water, containing 0.5 M NaCl, as supporting electrolyte, and then the voltammogram was recorded.

**Figure 4:**
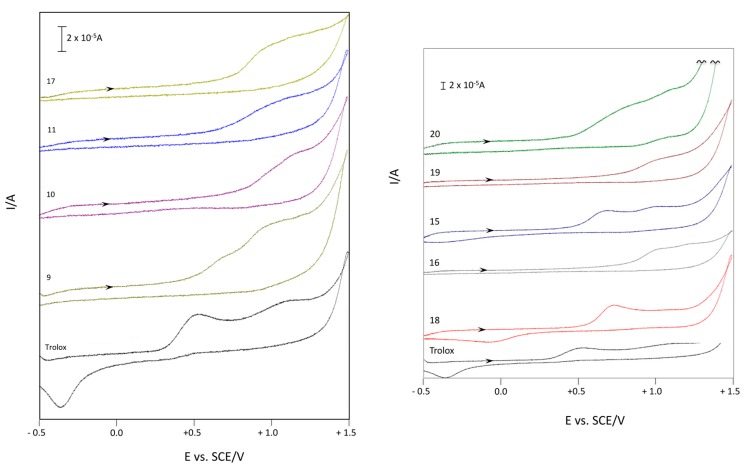
Voltammetric curves of compounds **9**–**11** and **15**–**20** (c = 2 × 10^−3^ M) in aqueous medium (H_2_O-0.5 M NaCl). Starting potential: −0.5 V; reversal potential: +1.5 V (GC working electrode, ν = 0.2 V·s^−1^, SCE reference electrode, 25 °C, N_2_).

As Figure 4 and Table 6 highlight, all the considered compounds showed a similar voltammetric behavior, i.e., an irreversible anodic oxidation whose E_p_^ox^ (oxidation peak potential) is between +0.62 and +1.13 V (vs SCE). All E_p_^ox^ of the considered products are of higher potential than that of the reference compound, Trolox (E_p_^ox^ 0.52 V vs. SCE), which corresponds to a minor easing of the oxidation process. Nevertheless, among these, products **9**, **15**, and **18** exhibited the lowest values of potential, which, being under 1V (E_p_^ox^ 0.72, 0.62, and 0.73 respectively) and quite close to the Trolox one, is indicative of antioxidant capacity. These results suggested that in these electrochemical measurements, the fundamental structural features necessary to increase the antioxidant capacity are the phenolic groups in positions 5 and 7 (in compounds **9**, **15**, and **18**, respectively). Moreover, the presence of a CF_3_ group seems essential for the ease in the oxidation process, lowering E_p_^ox^ by 60–120 mV (compare compound **9** vs. **15**, **10** vs. **16** and **11** vs. **17** in Table 6).

##### Cyclic Voltammetry in Acetonitrile of Compounds **9**–**11** and **15**–**20**

**Figure 5:**
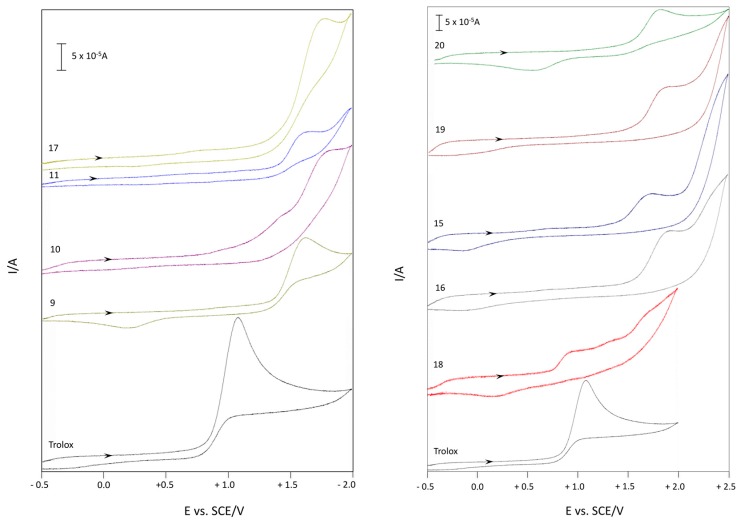
Voltammetric curves of compounds **9**–**11** and **15**–**20** in acetonitrile (c = 2 × 10^−3^ M) in acetonitrile (MeCN-0.1 M Et_4_NBF_4_). Starting potential: −0.5 V; reversal potential: +1.5 V (GC working electrode, ν = 0.2 V·s^−1^, SCE reference electrode, 25 °C, N_2_).

The CV in acetonitrile was performed in a cell containing a three-electrode system involving: (i) the reference electrode (modified SCE electrode, −0.029 V vs. SCE electrode− i.e., containing an organic junction); (ii) the working electrode (Glassy Carbon, GC); and (iii) a counter electrode (platinum wire). Each antioxidant (2 × 10^−3^ M final concentration), dissolved in ACN, was added to 5 mL of a solution of ACN, containing 0.1 M TEABF_4_ (tetraethylammonium tetrafluoroborate), as supporting electrolyte. The corresponding results are depicted in Figure 5 and Table 6.

As displayed in Table 6, the electrochemical results obtained from CV in acetonitrile were relatively in accord with those observed in aqueous medium. Specifically, compounds **9** and **10** showed lower potential (E_p_^ox^ 1.62 and 1.44, respectively), although they were higher than the value of Trolox (E_p_^ox^ 1.08 V). Remarkably, compound **18** exhibited the first potential peak at 0.92 V, lower than Trolox. This result showed that an essential structural feature which makes the 3-hydroxy-3Hbenzofuranone scaffold a possible antioxidant compound is the presence of phenolic group in *ortho* position and a CF_3_ group.

## 3. Materials and Methods

Solvents and common reagents were purchased from a commercial source and used without further purification. All reactions were monitored by thin-layer chromatography (TLC) carried out on Merck F-254 silica glass plates and visualized with UV light or by 5% phosphomolibdic acid/ethanol test. Flash chromatography was performed on Sigma-Aldrich silica gel (60, particle size: 0.040–0.063 mm). ^1^H-NMR and ^13^C-NMR were recorded in CDCl_3_ (99.8% in deuterium) using a Varian Gemini 300 spectrometer (300 MHz, Varian Inc., Palo Alto, CA, USA). All chemical shifts are expressed in parts per million (δ scale) and are referenced to the residual protons of the NMR solvent (CDCl_3_, δ 7.24 ppm). Coupling constant (*J*) was expressed in Hz. Infrared spectra (FT-IR) were obtained using a Bruker Vector 22 spectrometer (Bruker, Billerica, MA, USA); data are presented as the frequency of absorption (cm^−1^). High-resolution mass spectrometry (HRMS) spectra were recorded with Micromass Q-TOF micro mass spectrometer (Waters Corporations, Milford, MA, USA) and Micromass LCT (ESI, Waters Corporations, Milford, MA, USA) with Lock-Spray-Injector (Injection Loop-Modus in a HPLC system, Waters, Alliance 2695, Waters Corporations, Milford, MA, USA). UV-Vis measurements were performed with a Shimadzu-UV-2401PC spectrophotometer. Cyclic Voltammetry measurements were acquired on a AMEL552 electrochemical workstation. The standard three-electrode arrangement was employed. In all cases, a Pt wire auxiliary electrode was used, the working electrode was a 3 mm diameter glassy carbon, and the solution was degassed with N_2_. Melting points were determined on a Mel-Temp apparatus.

### 3.1. General Procedure for the Lewis-Acid-Catalysed Friedel–Crafts/Lactonization Reaction

The alkylating agent (2.2 mmol) was added in one portion to a stirred solution of the appropriate phenol (2.0 mmol) in anhydrous CHCl_3_ (9 mL), and then TiCl_4_ (1 M in anhydrous CH_2_Cl_2_; 0.4 mL, 10 mol-%) was added. The system was kept under an argon atmosphere. The clear reddish solution was stirred at the reported temperature until the substrate had been completely consumed (TLC monitoring). Afterwards, the reaction mixture was poured into cold water (18 mL), and the aqueous phase was extracted several times with EtOAc (4 × 20 mL). The combined organic layers were washed with brine, dried with anhydrous Na_2_SO_4_, and concentrated under vacuum. The residue was purified by flash chromatography on silica gel to give the corresponding products as described below.

### 3.2. Characterization Data for Benzofuran **9**–**11**

*Ethyl 3,5-Dihydroxy-2-oxo-2,3-Dihydrobenzofuran-3-Carboxylate,* **9**. Following the general procedure, the single product 9 was obtained as a white solid in 70% yield after purification by flash chromatography on silica gel (nHexane/EtOAc = 7/3). m.p. 139–142 °C. IR (CHCl_3_): $\tilde{\nu }$ = 3468–3412, 3018, 2979, 2914, 1759, 1725, 1608 cm^−1^. ^1^H-NMR (CDCl_3_, 300 MHz, 25 °C): δ (ppm) = 8.59 (bs, 1H, OH_phen_), 7.08 (d, *J* = 8.6 Hz, 1H, CH_arom_), 6.98–6.88 (m, 2H, CH_arom_), 6.50 (bs, 1H, OH), 4.30–4.15 (m, 2H, CH_2_CH_3_), 1.16 (t, *J* = 7.1 Hz, 3H, CH_2_CH_3_). ^13^C-NMR (CDCl_3_, 75 MHz, 25 °C): δ (ppm) 173.2, 168.5, 155.4, 147.7, 128.2, 118.6, 112.5, 111.7, 77.9, 63.3, 14.0. HRMS: exact mass calculated for (C_11_H_10_NaO_6_) requires *m*/*z* 261.0370, found *m*/*z* 261.0371.

*Ethyl 3,6-Dihydroxy-2-oxo-2,3-Dihydrobenzofuran-3-Carboxylate,* **10**. Following the general procedure, the single product **10** was obtained as a white solid in 62% yield after purification by flash chromatography on silica gel (*n*Hexane/EtOAc = 4/6). m.p. 142–144 °C. IR (CHCl_3_): $\tilde{\nu }$ = 3468–3420, 3010, 2972, 2921, 1760, 1727, 1615 cm^−1^. ^1^H-NMR (CDCl_3_, 300 MHz, 25 °C): δ (ppm) = 9.17 (bs, 1H, OH_phen_), 7.25 (d, *J* = 7.9 Hz, 1H, CH_arom_), 6.72 (m, 2H, CH_arom_), 6.37 (bs, 1H, OH), 4.29–4.08 (m, 2H, *CH_2_*CH_3_), 1.15 (t, *J* = 7.1 Hz, 3H, CH_2_*CH_3_*). ^13^C-NMR (CDCl_3_, 75 MHz, 25 °C): δ (ppm) 173.2, 168.7, 161.1, 155.9, 126.0, 118.0, 112.5, 99.5, 77.2, 63.0, 14.0. HRMS: exact mass calculated for (C_11_H_10_NaO_6_) requires *m*/*z* 261.0370, found *m*/*z* 261.0372.

*Ethyl 3,6-Dihydroxy-7-Methyl-2-oxo-2,3-Dihydrobenzofuran-3-Carboxylate,* **11**. Following the general procedure, the single product **11** was obtained as a white solid in 81% yield after purification by flash chromatography on silica gel (*n*Hexane/EtOAc = 4/6). m.p. 126–128 °C. IR (CHCl_3_): $\tilde{\nu }$ = 3460–3420, 3262, 3005, 2970, 2919, 1801, 1730, 1629 cm^−1^. ^1^H-NMR (CDCl_3_, 300 MHz, 25 °C): δ (ppm) = 8.32 (bs, 1H, OH_phen_), 7.02 (d, *J* = 7,9 Hz, 1H, CH_arom_), 6.61 (m, 2H, CH_arom_), 5.98 (bs, 1H, OH), 4.41–3.99 (m, 2H, *CH_2_*CH_3_), 2.15 (s, 3H, CCH_3_), 1.15 (t, *J* = 7.1 Hz, 3H, CH_2_*CH_3_*). ^13^C-NMR (CDCl_3_, 75 MHz, 25 °C): δ (ppm) 174.3, 169.7, 159.6, 154.9, 122.7, 117.8, 111.6, 109, 78.3, 63.5, 14.1, 9.6. HRMS: exact mass calculated for (C_12_H_12_NaO_6_) requires *m*/*z* 275.0526, found *m*/*z* 275.052.

### 3.3. General Procedure for the Domino Friedel-Crafts/Lactonization Reaction Performed in AcOH

The alkylating agent (5.5 mmol) was added in one portion to a stirred solution of the appropriate phenol (5.0 mmol) in acetic acid (3 mL). The system was kept under an argon atmosphere. The clear reddish solution was stirred at reflux temperature until the substrate had been completely consumed (TLC and HPLC monitoring). Afterwards, the reaction mixture was concentrated under vacuum and the residue was purified by flash chromatography on silica gel to give the products as described below.

### 3.4. Characterization Data for Benzofuran-2(3H)-One **15**–**20**

*3,5-Dihydroxy-3-(trifluoromethyl)benzofuran-2(3H)-One,* **15**. Following the general procedure, the single product **15** was obtained as a white solid in 35% yield after purification by flash chromatography on silica gel (*n*Hexane/Acetone = 8/2). m.p. 136–138 °C. IR (CHCl_3_): $\tilde{\nu }$ = 3460–3190, 3005, 2970, 2919, 1801, 1730, 1629, 1498 cm^−1^. ^1^H-NMR (CDCl_3_, 300 MHz, 25 °C): δ (ppm) = 8.72 (bs, 1H, OH_phen_), 7.24–6.95 (m, 4H, CH_arom_ + OH). ^13^C-NMR (CDCl_3_, 75 MHz, 25 °C): δ (ppm) 168.7, 153.8, 145.7, 121.8, 121.6 (q, ^1^*J*_CF_ = 283.3 Hz), 118.1, 111.4, 111.0, 74.0 (q, ^2^*J*_CF_ = 32.5 Hz). HRMS: exact mass calculated for (C_9_H_5_NaF_3_O_4_) requires *m*/*z* 257.0032, found *m*/*z* 257.0033.

*3,6-Dihydroxy-3-(trifluoromethyl)benzofuran-2(3H)-One,* **16**. Following the general procedure, the single product **16** was obtained as a white solid in 28% yield after purification by flash chromatography on silica gel (*n*Hexane/Et_2_O = 1/1). m.p. 138–140 °C. IR (CHCl_3_): $\tilde{\nu }$ = 3490–3230, 3010, 2970, 2923, 1806, 1735, 1637 cm^−1^. ^1^H-NMR (CDCl_3_, 300 MHz, 25 °C): δ (ppm) = 9.40 (bs, 1H, OH_phen_), 7.45 (d, *J* = 9 Hz, 2H, CH_arom_), 7.02 (bs, 1H, OH), 6.80 (d, *J* = 9 Hz, 1H, CH_arom_), 6.74 (s, 1H, CH_arom_). ^13^C-NMR (CDCl_3_, 75 MHz, 25 °C): δ (ppm) 170.5, 162.1, 155.9, 127.7, 123.6 (q, ^1^*J*_CF_ = 283.9 Hz), 113.2, 113.0, 99.5, 75.2 (q, ^2^*J*_CF_ = 32.8 Hz). HRMS: exact mass calculated for (C_9_H_5_NaF_3_O_4_) requires *m*/*z* 257.0032, found *m*/*z* 257.0031.

*3,6-Dihydroxy-7-methyl-3-(trifluoromethyl)benzofuran-2(3H)-One,* **17***.* Following the general procedure, the single product **17** was obtained as a white solid in 61% yield after purification by flash chromatography on silica gel (*n*Hexane/Et_2_O=1/1). m.p. 129–131 °C. IR (CHCl_3_): $\tilde{\nu }$ = 3470–3220, 3000, 2975, 2913, 1826, 1724, 1630 cm^−1^. ^1^H-NMR (CDCl_3_, 300 MHz, 25 °C): δ (ppm) = 7.32 (bs, 1H, OH_phen_), 7.08 (d, *J* = 8.4 Hz, 1H, CH_arom_), 6.58 (d, *J* = 8.3 Hz, 1H, CH_arom_), 4.78 (bs, 1H, OH), 2.22 (s, 3H, C*CH_3_*). ^13^C-NMR (CDCl_3_, 75 MHz, 25 °C): δ (ppm) 170.5, 159.3, 153.4, 123.2, 122.8 (q, ^1^*J*_CF_ = 283.8 Hz), 112.1, 111.6, 108.6, 75.2 (q, ^2^*J*_CF_ = 32.8 Hz), 8.12. HRMS: exact mass calculated for (C_10_H_7_NaF_3_O_4_) requires *m*/*z* 271.0189, found *m*/*z* 271.0190.

*3,7-Dihydroxy-3-(trifluoromethyl)benzofuran-2(3H)-One,* **18**. Following the general procedure, the single product **18** was obtained as a white solid in 78% yield after purification by flash chromatography on silica gel (*n*Hexane/Et_2_O = 1/1). m.p. 142–144 °C. IR (CHCl_3_): $\tilde{\nu }$ = 3498–3256, 3012, 2988, 2909, 1831, 1744, 1629 cm^−1^. ^1^H-NMR (CDCl_3_, 300 MHz, 25 °C): δ (ppm) = 9.20 (bs, 1H, OH_fen_), 7.23–7.07 (m, 4H, CH_arom_ + OH).^13^C-NMR (CDCl_3_, 75 MHz, 25 °C): δ (ppm) 169.2, 141.6, 125.9, 123.6, 122.8 (q, ^1^*J*_CF_ = 284.1 Hz), 120.3, 119.8, 116.4, 75.0 (q, ^2^*J*_CF_ = 32.6 Hz). HRMS: exact mass calculated for (C_9_H_5_NaF_3_O_4_) requires *m*/*z* 257.0032, found *m*/*z* 257.0032.

*3,4,6-Trihydroxy-3-(trifluoromethyl)benzofuran-2(3H)-One,* **19**. Following the general procedure, the single product **19** was obtained as a white solid in 66% yield after purification by flash chromatography on silica gel (*n*Hexane/Acetone=7/3). m.p. 174–176 °C. IR (CHCl_3_): $\tilde{\nu }$ = 3500–3280, 3023, 3000, 2959, 1821, 1754, 1607 cm^−1^. ^1^H-NMR (CDCl_3_, 300 MHz, 25 °C): δ (ppm) = 9.30 (bs, 1H, OH_fen_), 9.22 (bs, 1H, OH_phen_), 6.71 (bs, 1H, OH_fen_), 6.28 (s, 1H, CH_arom_), 6.23 (s, 1H, CH_arom_).^13^C-NMR (CDCl_3_, 75 MHz, 25 °C): δ (ppm) 167.3, 162.0, 159.4, 158.0, 123.2 (q, ^1^*J*_CF_ = 285.7 Hz), 122.7, 98.4, 95.6, 75.4 (q, ^2^*J*_CF_ = 33.6 Hz). HRMS: exact mass calculated for (C_9_H_5_NaF_3_O_5_) requires *m*/*z* 272.9981, found *m*/*z* 272.9983.

*3,6,7-Trihydroxy-3-(trifluoromethyl)benzofuran-2(3H)-One,* **20**. Following the general procedure, the single product **20** was obtained as a white solid in 76% yield after purification by flash chromatography on silica gel (*n*Hexane/Et_2_O = 3/7). m.p. 148–150 °C. IR (CHCl_3_): $\tilde{\nu }$ = 3517–3239, 3045, 3018, 2960, 1819, 1734, 1615 cm^−1^. ^1^H-NMR (CDCl_3_, 300 MHz, 25 °C): δ (ppm) = 9.03 (bs, 1H, OH_phen_), 8.55 (bs, 1H, OH_phen_), 6.95 (d, *J =* 8.4 Hz, 1H, CH_arom_), 6.81 (d, *J =* 8.4 Hz, 1H, CH_arom_), 6.65 (bs, 1H, OH).^13^C-NMR (CDCl_3_, 75 MHz, 25 °C): δ (ppm) 169.7, 149.6, 142.0, 130.0, 122.9 (q, ^1^*J*_CF_ = 284.0 Hz), 116.6, 113.8, 111.9, 75.0 (q, ^2^*J*_CF_ = 35.1 Hz). HRMS: exact mass calculated for (C_9_H_5_NaF_3_O_5_) requires *m*/*z* 272.9981, found *m*/*z* 272.9980.

## 4. Conclusions

In summary, we have synthesized a series of 3,3-disubstituted-3*H*-benzofuran-2-one derivatives (**9**–**11** and **15**–**20**) by improving our previous findings concerning the domino Friedel–Crafts/lactonization reaction. With respect the chosen electrophile as well as the various polyphenols (**1**–**6**) used as nucleophilic counterpart, two different protocols were followed: (i) the use of TiCl_4_ as catalyst in CHCl_3_ at 60 °C; and (ii) the employment of AcOH as solvent at 120 °C without the addition of any further catalyst. Afterwards, the antioxidant capacity of the synthesized compounds (**9**–**11** and **15**–**20**) was evaluated using DPPH assay and Cyclic Voltammetry, performing the experiments in both MeOH and acetonitrile. Specifically, the benzofuran-2-ones **9**, **15**, **18**, and **20** presented the best values of rIC_50_. Among them, compound **20** exhibited a remarkable rIC_50_ of 0.18 (MeOH) and 0.17 (ACN), and therefore possessed the greatest antioxidant activity by reducing three molecules of DPPH^•^ in both solvents, a result that is validated despite the possible interference of the solvent which was evaluated and excluded almost completely by measuring the rate constants for the reaction with DPPH^•^. Additionally, the electrochemical measurements (CV), recorded in in aqueous medium as well as in acetonitrile, established compounds **18** and **20** as the best reducing agents among the tested molecules. The observed results undeniably suggest that the 3-hydroxy-benzofuran-2-one scaffold should be involved in the antioxidant mechanism: the presence of a hydroxyl group at C-7 position as well as a strong electron withdrawing group at C-3 position are the essential structural features. Detailed additional in vitro tests to further endorse the notable and promising antioxidant activity of the studied compounds are ongoing in our laboratories and will be reported in due course.

## Figures and Tables

**Table 1:** Friedel–Crafts alkylation/lactonization of polyphenols **1**–**6** performed with TiCl_4_ as catalyst *^a^*.

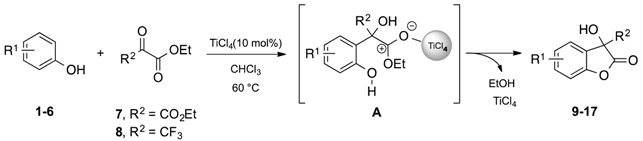						
Entry	Substrate		R^2^	Product		Yield (%) *^b^*
1	**1**	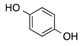	CO_2_Et	**9**	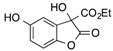	70
2	**2**	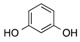	CO_2_Et	**10**	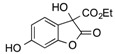	62
3	**3**	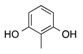	CO_2_Et	**11**	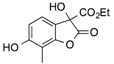	81
4	**4**	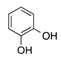	CO_2_Et	**12**	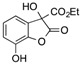	4 (22 *^c^*)
5	**5**	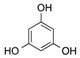	CO_2_Et	**13**	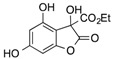	7 (18 *^c^*)
6	**6**	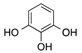	CO_2_Et	**14**	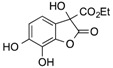	3 (19 *^c^*)
7	**1**	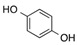	CF_3_	**15**	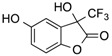	35
8	**2**	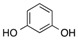	CF_3_	**16**	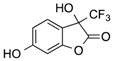	28
9	**3**	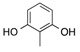	CF_3_	**17**	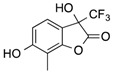	61
10	**4**	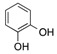	CF_3_	**18**	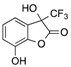	5
11	**5**	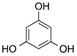	CF_3_	**19**	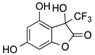	11
12	**6**	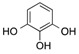	CF_3_	**20**	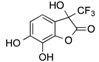	7

*^a^* Unless otherwise stated, the reactions were performed with indicated phenols (2.0 mmol) and alkylating agents (**7** or **8**; 2.2 mmol) in the presence of TiCl_4_ (10 mol%) in anhydrous CHCl_3_ (9 mL); *^b^* Yield of the isolated product; *^c^* Yield calculated at the ^1^H-NMR of the crude reaction mixture.

**Table 2:** Reaction of 3,3,3-trifluoromethyl pyruvate **8** with polyphenols **1–6** *^a^*.

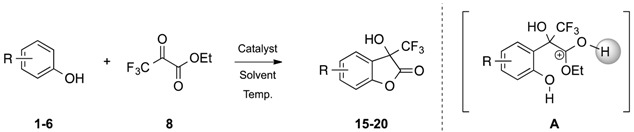								
Entry	Sub	Catalyst	mol%	Solvent	Temp (°C)	Time (h)	Product	Yield (%) *^b^*
1	**1**	BF_3_·Et_2_O	30	CH_2_Cl_2_	r.t	48	**15**	5
2	**1**	BF_3_·Et_2_O	30	THF	60	22	**15**	n.d.
3	**1**	AlCl_3_	30	CH_2_Cl_2_	r.t.	72	**15**	n.d.
4	**1**	PhCO_2_H	30	CHCl_3_	60	72	**15**	n.d.
5	**1**	PhCO_2_H	100	CHCl_3_	60	24	**15**	n.d.
6	**1**	CSA	30	CHCl_3_	60	24	**15**	n.d.
7	**1**	CSA	100	Toluene	100	10	**15**	n.d.
8	**1**	DBU	30	CH_2_Cl_2_	r.t.	72	**15**	n.d.
9	**1**	AcOH	30	CH_2_Cl_2_	r.t.	72	**15**	n.d.
10	**1**	-	-	AcOH	120	12	**15**	45
11 *^c^*	**1**	-	-	AcOH	120	10	**15**	55
12 *^d^*	**1**	-	-	AcOH	120	4	**15**	80
13 *^d^*	**2**	-	-	AcOH	120	4	**16**	72
14 *^d^*	**3**	-	-	AcOH	120	6	**17**	86
15 *^d^*	**4**	-	-	AcOH	120	4	**18**	78
16 *^d^*	**5**	-	-	AcOH	120	5	**19**	66
17 *^d^*	**6**	-	-	AcOH	120	4	**20**	76

*^a^* Unless otherwise stated, the reactions were performed under inert atmosphere with the indicated phenols (5.0 mmol) and 3,3,3-trifluoromethyl pyruvate (**8**; 5.5 mmol) in 8 mL of solvent; *^b^* Yield of the isolated product. (n.d. = not detected); *^c^* 5 mL of AcOH was employed; *^d^* 3 mL of AcOH was employed.

**Table 3:** Antioxidant capacity of compounds **9**–**11** and **15**–**20** towards DPPH^•^ in methanol ^a^.

Entry	Antioxidant	rIC_50_ (mols_antiox.t_/mols_DPPH__∙__)_	N° DPPH^•^ Reduced ^b^
1	Trolox	0.23	2.16
2	9	0.31	1.63
3	10	3.62	0.14
4	11	2.03	0.25
5	15	0.22	2.28
6	16	3.52	0.14
7	17	1.07	0.47
8	18	0.24	2.07
9	19	0.62	0.80
10	20	0.18	2.72

^a^ All the measures were performed in triplicate and the values were reported as mean ± SD; ^b^ The number of DPPH^•^ molecules, reduced by one molecule of antioxidant, is the inverse of the stoichiometry.

**Table 4:** Antioxidant capacity of compounds **9**–**11** and **15**–**20** towards DPPH^•^ in acetonitrile ^a^.

Entry	Antiox.	rIC_50_ (mols_antiox._/mols_DPPH__∙_)	N° DPPH^•^ Reduced ^b^
1	Trolox	0.22	2.24
2	9	4.26	0.12
3	10	4.12	0.12
4	11	3.92	0.13
5	15	1.69	0.30
6	16	4.47	0.11
7	17	3.25	0.15
8	18	0.54	0.93
9	19	2.23	0.22
10	20	0.17	3.02

^a^ All the measures were performed in triplicate and the values were reported as mean ± SD; ^b^ The number of DPPH^•^ molecules, reduced by one molecule of antioxidant, is the inverse of the stoichiometry.

**Table 5:** Bimolecular rate constants (M^−1^·s^−1^) for H-atom abstraction from compounds **9**, **15**, **18**, and **20** by DPPH^•^ *^a^*.

Entry	Antiox.	k^s^ (M^−1^·s^−1^) *^b^*	rIC_50_ (mols_antioxidant_/mols_DPPH•_)	k^s^ (M^−1^·s^−1^) *^b^*	rIC_50_ (mols_antioxidant_/mols_DPPH•_)
		MeOH	MeOH	ACN	ACN
1	9	3.26	0.31	8.54	4.26
2	15	3.77	0.22	1.26	1.69
3	18	1.40	0.24	0.23	0.54
4	20	0.77	0.18	2.22	0.17

*^a^* All the measurements were performed in triplicate and processed using a *Sigma Plot* software, 12.0, Systat software, Inc., San Jose, CA, USA); *^b^* The second-order rate constants were the slopes of the plots of k_es_ vs. compound concentration (See Supplementary Material).

**Table 6:** First oxidation peaks (E_p_^ox^) from CV in aqueous medium and acetonitrile relative to compounds **9**–**11** and **15**–**20** (Figure 3 and Figure 4).

Entry	Compounds	E_p_^ox^ (V) ^1^ (H_2_O)	E_p_^ox^ (V) ^1^ (ACN)
1	Trolox	0.52	1.08
2	**9**	0.72	1.62
3	**10**	1.13	1.44
4	**11**	1.11	1.65
5	**15**	0.62	1.72
6	**16**	1.01	1.92
7	**17**	1.05	1.77
8	**18**	0.73	0.92
9	**19**	1.03	1.88
10	**20**	0.85	1.81

^1^ All the peak potentials are referred to SCE.

## Supplementary Materials

The following are available online at http://www.mdpi.com/1420-3049/23/4/710/s1. Experimental procedures, characterization data of synthesized compounds, explanation of the reaction mechanism, and the complete set of antioxidant assay are reported in the Supplementary Material.
