# Supplementary material for: Synthesis of Benzofuran-2-One Derivatives and Evaluation of Their Antioxidant Capacity by Comparing DPPH Assay and Cyclic Voltammetry
Source: Molecules. 2018 Mar 21;23(4):710. doi: 10.3390/molecules23040710 (PMC6017620; doi:10.3390/molecules23040710)
Supplement: Supplementary file 1 [file molecules-23-00710-s001.pdf]

Supplementary Material

for

Synthesis of benzofuran-2-one derivatives and evaluation of their antioxidant capacity by comparing DPPH assay and Cyclic Voltammetry

Martina Miceli [1,*], Elia Roma [1], Paolo Rosa [2], Marta Feroci [3], M. Antonietta Loreto [4], Daniela Tofani [1], and Tecla Gasperi [1,*]

[1] Dipartimento di Scienze, Sezione di Nanoscienze e Nanotecnologie, Università degli Studi di Roma Tre, via della Vasca Navale 79, I-00146, Roma, Italy; martina.miceli@icloud.com (M.M.); elia.roma@uniroma3.it (E.R.); daniela.tofani@uniroma3.it; (D.T.); tecla.gasperi@uniroma3.it (T.G.)

[2] Dipartimento di Scienze e Biotecnologie Medico-Chirurgiche, Sapienza Università di Roma – Polo Pontino, Corso della Repubblica 79, 04100, Latina, Italy; p.rosa@uniroma1.it (P.R.)

[3] Dipartimento di Scienze di Base e Applicate per l'Ingegneria, Sapienza Università di Roma, via Castro Laurenziano 7, I-00161 Roma, Italia; marta.feroci@uniroma1.it (M.F.)

[4] Dipartimento di Chimica, Sapienza Università di Roma, p.le A. Moro 5, I-00185 Roma, Italia; mariaantonietta.loreto@uniroma3.it (M.A.L.)

* Correspondence: tecla.gasperi@uniroma3.it; Tel.: +39-3386711045

Received: date; Accepted: date; Published: date

Abstract: The present work aims to synthesise promising antioxidant compounds as valuable alternative to the nowadays too expensive and easily degradable molecules that are employed as stabilizers in industrial preparation. Taking into account our experience concerning domino Friedel-Crafts/lactonization reactions, we successfully improved and extended the previously reported methodology toward the synthesis of 3,3-disubstituted-3H-benzofuran-2-one derivatives **9**-**20** starting from polyphenols **1-6** as substrates and either diethylketomalonate (**7**) or 3,3,3-trifluoromethyl pyruvate (**8**) as electrophilic counterpart .The antioxidant capacity of the most stable compounds (**9-11** and **15-20**) was evaluated by both DPPH assay and Cyclic Voltammetry analyses performed in alcoholic media (methanol) as well as in aprotic solvent (acetonitrile). By comparing the recorded experimental data, a remarkable activity can be attributed to few of the tested lactones.

Keywords: antioxidant activity; cyclic voltammetry; DPPH; domino reaction; benzofuran-2-one.

1. General Information....3

2. Syntheses of 3-ylideneoxindoles 9-11 3

2.1 General Procedure for the Lewis-acid-catalysed Friedel-Crafts/lactonization reaction 3

2.2. Characterization Data for benzofuran 9-11 3

Ethyl 3,5-dihydroxy-2-oxo-2,3-dihydrobenzofuran-3-carboxylate, ***9*** 3

Ethyl 3,6-dihydroxy-2-oxo-2,3-dihydrobenzofuran-3-carboxylate, ***10*** 3

Ethyl 3,6-dihydroxy-7-methyl-2-oxo-2,3-dihydrobenzofuran-3-carboxylate, ***11*** 4

2.3 General Procedure for the acid-catalysed Friedel-Crafts/lactonization reaction 4

2.4. Characterization Data for benzofuran 15-20 4

3,5-Dihydroxy-3-(trifluoromethyl)benzofuran-2(3H)-one, ***15*** 4

3,6-Dihydroxy-3-(trifluoromethyl)benzofuran-2(3H)-one, ***16*** 4

3,6-Dihydroxy-7-methyl-3-(trifluoromethyl)benzofuran-2(3H)-one, ***17***....4

3,7-Dihydroxy-3-(trifluoromethyl)benzofuran-2(3H)-one, ***18*** 5

3,4,6-Trihydroxy-3-(trifluoromethyl)benzofuran-2(3H)-one, ***19***....5

3,6,7-Trihydroxy-3-(trifluoromethyl)benzofuran-2(3H)-one, ***20***....5

3. DPPH assay 6

3.1. Calibration curves 6
3.2. DPPH assay in methanol 6
3.3. DPPH assay in acetonitrile 8
3.4. Measurements of Rate Constants for the reaction of compounds 9, 15, 18, and 20 with DPPH$^•$ 9
References 11

1. General Information

Solvents and common reagents were purchased from a commercial source and used without further purification. All reactions were monitored by thin-layer chromatography (TLC) carried out on Merck F-254 silica glass plates and visualized with UV light or by 5% phosphomolibdic acid/ethanol test. Flash chromatography was performed on Sigma-Aldrich silica gel (60, particle size: 0.040–0.063 mm). ^{1}H-NMR and ^{13}C-NMR were recorded in $CDCl_3$ (99.8% in deuterium) using a Varian Gemini 300 spectrometer (300 MHz, Varian inc., Palo Alto, CA, USA). All chemical shifts are expressed in parts per million (δ scale) and are referenced to the residual protons of the NMR solvent ($CDCl_3$, δ 7.24 ppm). Coupling constant (*J*) was expressed in Hz. Infrared spectra (FT-IR) were obtained using a Bruker Vector 22 spectrometer (Bruker, Billerica, MA, USA); data are presented as the frequency of absorption (cm^{-1}). High-resolution mass spectrometry (HRMS) spectra were recorded with Micromass Q-TOF micro mass spectrometer (Waters Corporations, Milford, MA, USA) and Micromass LCT (ESI, Waters Corporations, Milford, MA, USA) with Lock-Spray-Injector (Injection Loop-Modus in a HPLC system, Waters, Alliance 2695). UV-Vis measurements were performed with a Shimadzu-UV-2401PC spectrophotometer. Cyclic Voltammetry measurements were acquired on a AMEL552 electrochemical workstation. The standard three-electrode arrangement was employed. In all case, a Pt wire auxiliary electrode was used, the working electrode was a 3 mm diameter glassy carbon, and the solution was degassed with N_2. Melting points were determined on a Mel-Temp apparatus.).

2. Syntheses of 3-ylideneoxindoles 9-11

2.1 General Procedure for the Lewis-acid-catalysed Friedel-Crafts/lactonization reaction

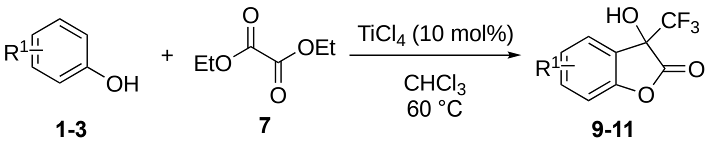

1-3 **7** **9-11**

The alkylating agent (2.2 mmol) was added in one portion to a stirred solution of the appropriate phenol (2.0 mmol) in anhydrous $CHCl_3$ (9 mL), and then $TiCl_4$ (1 M in anhydrous CH_2Cl_2; 0.4 mL, 10 mol-%) was added. The system was kept under an argon atmosphere. The clear reddish solution was stirred at the reported temperature until the substrate had been completely consumed (TLC monitoring). Afterwards, the reaction mixture was poured into cold water (18 mL), and the aqueous phase was extracted several times with EtOAc (4 x 20 mL). The combined organic layers were washed with brine, dried with anhydrous Na_2SO_4, and concentrated under vacuum. The residue was purified by flash chromatography on silica gel to give the products as described below.

*2.2. Characterization Data for benzofuran **9-11***

*Ethyl 3,5-dihydroxy-2-oxo-2,3-dihydrobenzofuran-3-carboxylate, **9***

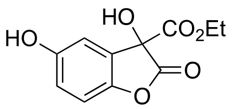

Following the general procedure, the single product 9 was obtained as a white solid in 70% yield after purification by flash chromatography on silica gel (nHexane/EtOAc=7/3). m.p. 139-142 °C. IR ($CHCl_3$): $\tilde{\nu}$= 3468 – 3412, 3018, 2979, 2914, 1759, 1725, 1608 cm^{-1}. ^{1}H NMR ($CDCl_3$, 300MHz, 25 °C): δ (ppm) = 8.59 (bs, 1H, OH_{phen}), 7.08 (d, *J* =8.6 Hz, 1H, CH_{arom}), 6.98 – 6.88 (m, 2H, CH_{arom}), 6.50 (bs, 1H, OH), 4.30 – 4.15 (m, 2H, CH_2CH_3), 1.16 (t, *J* = 7.1 Hz, 3H, CH_2CH_3). ^{13}C NMR ($CDCl_3$, 75MHz, 25 °C): δ (ppm) 173.2, 168.5, 155.4, 147.7, 128.2, 118.6, 112.5, 111.7, 77.9, 63.3, 14.0. HRMS: exact mass calculated for ($C_{11}H_{10}NaO_6$) requires *m*/*z* 261.0370, found *m*/*z* 261.0371.

*Ethyl 3,6-dihydroxy-2-oxo-2,3-dihydrobenzofuran-3-carboxylate, **10***

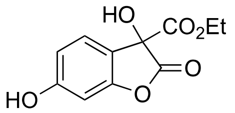

Following the general procedure, the single product **10** was obtained as a white solid in 62% yield after purification by flash chromatography on silica gel (*n*Hexane/EtOAc=4/6). m.p. 142-144 °C. IR ($CHCl_3$): $\tilde{\nu}$= 3468 – 3420, 3010, 2972, 2921, 1760, 1727, 1615 cm^{-1}. ^{1}H NMR ($CDCl_3$, 300MHz, 25 °C): δ (ppm) = 9.17 (bs, 1H, OH_{phen}), 7.25 (d, *J* =7,9 Hz, 1H, CH_{arom}), 6.72 (m, 2H, CH_{arom}), 6.37 (bs, 1H, OH), 4.29 – 4.08 (m, 2H, CH_2CH_3), 1.15 (t, *J* = 7.1

Hz, 3H, CH_2CH_3). ^{13}C NMR ($CDCl_3$, 75MHz, 25 °C): δ (ppm) 173.2, 168.7, 161.1, 155.9, 126.0, 118.0, 112.5, 99.5, 77.2, 63.0, 14.0. HRMS: exact mass calculated for ($C_{11}H_{10}NaO_6$) requires *m*/*z* 261.0370, found *m*/*z* 261.0372.

Ethyl 3,6-dihydroxy-7-methyl-2-oxo-2,3-dihydrobenzofuran-3-carboxylate, ***11***

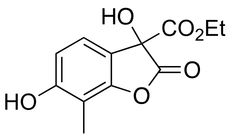

Following the general procedure, the single product **11** was obtained as a white solid in 81% yield after purification by flash chromatography on silica gel (*n*Hexane/EtOAc=4/6). m.p. 126-128 °C. IR ($CHCl_3$): $\tilde{\nu}$= 3460 – 3420, 3262, 3005, 2970, 2919, 1801, 1730, 1629 cm^{-1}. ^{1}H NMR ($CDCl_3$, 300MHz, 25 °C): δ (ppm) = 8.32 (bs, 1H, OH_{phen}), 7.02 (d, *J* =7,9 Hz, 1H, CH_{arom}), 6.61 (m, 2H, CH_{arom}), 5.98 (bs, 1H, OH), 4.41 – 3.99 (m, 2H, CH_2CH_3), 2.15 (s, 3H, CCH_3), 1.15 (t, *J* = 7.1 Hz, 3H, CH_2CH_3). ^{13}C NMR ($CDCl_3$, 75MHz, 25 °C): δ (ppm) 174.3, 169.7, 159.6, 154.9, 122.7, 117.8, 111.6, 109., 78.3, 63.5, 14.1, 9.6. HRMS: exact mass calculated for ($C_{12}H_{12}NaO_6$) requires *m*/*z* 275.0526, found *m*/*z* 275.052.

2.3 General Procedure for the acid-catalysed Friedel-Crafts/lactonization reaction

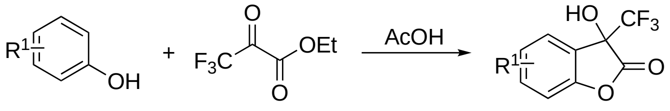

1-3 **8** **15-20**

The alkylating agent (5.5 mmol) was added in one portion to a stirred solution of the appropriate phenol (5.0 mmol) in acetic acid (3 mL). The system was kept under an argon atmosphere. The clear reddish solution was stirred at reflux temperature, until the substrate had been completely consumed (TLC and HPLC monitoring). Afterwards, the reaction mixture was concentrated under vacuum and the residue was purified by flash chromatography on silica gel to give the products as described below.

2.4. Characterization Data for benzofuran 15-20

3,5-Dihydroxy-3-(trifluoromethyl)benzofuran-2(3H)-one, ***15***

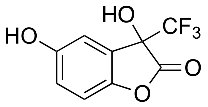

Following the general procedure, the single product **15** was obtained as a white solid in 35% yield after purification by flash chromatography on silica gel (*n*Hexane/Acetone=8/2). m.p. 136-138 °C. IR ($CHCl_3$): $\tilde{\nu}$= 3460 – 3190, 3005, 2970, 2919, 1801, 1730, 1629, 1498 cm^{-1}. ^{1}H NMR ($CDCl_3$, 300MHz, 25 °C): δ (ppm) = 8.72 (bs, 1H, OH_{phen}), 7.24 – 6.95 (m, 4H, CH_{arom} + OH). ^{13}C NMR ($CDCl_3$, 75MHz, 25 °C): δ (ppm) 168.7, 153.8, 145.7, 121.8, 121.6 (q, $^1J_{CF}$ = 283.3 Hz), 118.1, 111.4, 111.0, 74.0 (q, $^2J_{CF}$ = 32.5 Hz). HRMS: exact mass calculated for ($C_9H_5NaF_3O_4$) requires m/z 257.0032, found m/z 257.0033.

3,6-Dihydroxy-3-(trifluoromethyl)benzofuran-2(3H)-one, ***16***

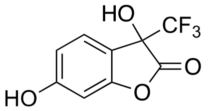

Following the general procedure, the single product **16** was obtained as a white solid in 28% yield after purification by flash chromatography on silica gel (*n*Hexane/Et_2O=1/1). m.p. 138-140 °C. IR ($CHCl_3$): $\tilde{\nu}$= 3490 – 3230, 3010, 2970, 2923, 1806, 1735, 1637 cm^{-1}. ^{1}H NMR ($CDCl_3$, 300MHz, 25 °C): δ (ppm) = 9.40 (bs, 1H, OH_{phen}), 7.45 (d, *J* =9 Hz, 2H, CH_{arom}), 7.02 (bs, 1H, OH), 6.80 (d, *J* =9 Hz, 1H, CH_{arom}), 6.74 (s, 1H, CH_{arom}). ^{13}C NMR ($CDCl_3$, 75MHz, 25 °C): δ (ppm) 170.5, 162.1, 155.9, 127.7, 123.6 (q, $^1J_{CF}$ = 283.9 Hz), 113.2, 113.0, 99.5, 75.2 (q, $^2J_{CF}$ =32.8 Hz). HRMS: exact mass calculated for ($C_9H_5NaF_3O_4$) requires *m*/*z* 257.0032, found *m*/*z* 257.0031.

3,6-Dihydroxy-7-methyl-3-(trifluoromethyl)benzofuran-2(3H)-one, ***17***

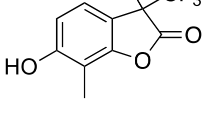

Following the general procedure, the single product **17** was obtained as a white solid in 61% yield after purification by flash chromatography on silica gel (*n*Hexane/Et_2O=1/1). m.p. 129-131 °C. IR ($CHCl_3$): $\tilde{\nu}$= 3470 – 3220, 3000, 2975, 2913, 1826, 1724, 1630 cm^{-1}. ^{1}H NMR ($CDCl_3$, 300MHz, 25 °C): δ (ppm) = 7.32 (bs, 1H, OH_{phen}), 7.08 (d, *J* =8.4 Hz, 1H, CH_{arom}), 6.58 (d, *J* =8.3 Hz, 1H, CH_{arom}), 4.78 (bs, 1H, OH), 2.22 (s, 3H, CCH_3). ^{13}C NMR

($CDCl_3$, 75MHz, 25 °C): δ (ppm) 170.5, 159.3, 153.4, 123.2, 122.8 (q, ${}^1J_{CF}$ = 283.8 Hz), 112.1, 111.6, 108.6, 75.2 (q, ${}^2J_{CF}$ =32.8 Hz), 8.12. HRMS: exact mass calculated for ($C_{10}H_7NaF_3O_4$) requires *m/z* 271.0189, found m/z 271.0190.

3,7-Dihydroxy-3-(trifluoromethyl)benzofuran-2(3H)-one, ***18***

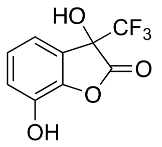

Following the general procedure, the single product **18** was obtained as a white solid in 78% yield after purification by flash chromatography on silica gel (*n*Hexane/Et_2O=1/1). m.p. 142-144 °C. IR ($CHCl_3$): $\tilde{\nu}$= 3498 – 3256, 3012, 2988, 2909, 1831, 1744, 1629 cm^{-1}. ${}^1H$ NMR ($CDCl_3$, 300MHz, 25 °C): δ (ppm) = 9.20 (bs, 1H, OH_{fen}), 7.23 – 7.07 (m, 4H, CH_{arom} + OH).${}^{13}C$ NMR ($CDCl_3$, 75MHz, 25 °C): δ (ppm) 169.2, 141.6, 125.9, 123.6, 122.8 (q, ${}^1J_{CF}$ = 284.1 Hz), 120.3, 119.8, 116.4, 75.0 (q, ${}^2J_{CF}$ =32.6 Hz). HRMS: exact mass calculated for ($C_9H_5NaF_3O_4$) requires *m/z* 257.0032, found *m/z* 257.0032.

3,4,6-Trihydroxy-3-(trifluoromethyl)benzofuran-2(3H)-one, ***19***

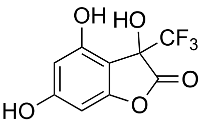

Following the general procedure, the single product **19** was obtained as a white solid in 66% yield after purification by flash chromatography on silica gel (*n*Hexane/Acetone=7/3). m.p. 174-176 °C. IR ($CHCl_3$): $\tilde{\nu}$= 3500 – 3280, 3023, 3000, 2959, 1821, 1754, 1607 cm^{-1}. ${}^1H$ NMR ($CDCl_3$, 300MHz, 25 °C): δ (ppm) = 9.30 (bs, 1H, OH_{fen}), 9.22 (bs, 1H, OH_{phen}), 6.71 (bs, 1H, OH_{fen}), 6.28 (s, 1H, CH_{arom}), 6.23 (s, 1H, CH_{arom}).${}^{13}C$ NMR ($CDCl_3$, 75MHz, 25 °C): δ (ppm) 167.3, 162.0, 159.4, 158.0, 123.2 (q, ${}^1J_{CF}$ = 285.7 Hz), 122.7, 98.4, 95.6, 75.4 (q, ${}^2J_{CF}$ =33.6 Hz). HRMS: exact mass calculated for ($C_9H_5NaF_3O_5$) requires *m/z* 272.9981, found *m/z* 272.9983.

3,6,7-Trihydroxy-3-(trifluoromethyl)benzofuran-2(3H)-one, ***20***

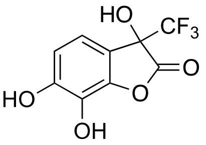

Following the general procedure, the single product **20** was obtained as a white solid in 76% yield after purification by flash chromatography on silica gel (*n*Hexane/Et_2O=3/7). m.p. 148-150 °C. IR ($CHCl_3$): $\tilde{\nu}$= 3517 – 3239, 3045, 3018, 2960, 1819, 1734, 1615 cm^{-1}. ${}^1H$ NMR ($CDCl_3$, 300MHz, 25 °C): δ (ppm) = 9.03 (bs, 1H, OH_{phen}), 8.55 (bs, 1H, OH_{phen}), 6.95 (d, *J* = 8.4 Hz, 1H, CH_{arom}), 6.81 (d, *J* = 8.4 Hz, 1H, CH_{arom}), 6.65 (bs, 1H, OH).${}^{13}C$ NMR ($CDCl_3$, 75MHz, 25 °C): δ (ppm) 169.7, 149.6, 142.0, 130.0, 122.9 (q, ${}^1J_{CF}$ = 284.0 Hz), 116.6, 113.8, 111.9, 75.0 (q, ${}^2J_{CF}$ =35.1 Hz). HRMS: exact mass calculated for ($C_9H_5NaF_3O_5$) requires *m/z* 272.9981, found *m/z* 272.9980.

3. DPPH assay

3.1. Calibration curves

From a stock solution of 250 μM DPPH• in MeOH or ACN, previously degassed with a N_2 flow, ten standards at different concentrations from 0 to 110 μM were prepared. The absorbance (λ_{max} = 517 nm) for each standard was measured in a Cary300 spectrophotometer or Shimadzu-UV-2401PC spectrophotometer and plotted versus the concentration of DPPH•. Figure SM-1 and Figure SM-2 show the calibration curves in methanol and acetonitrile, respectively.

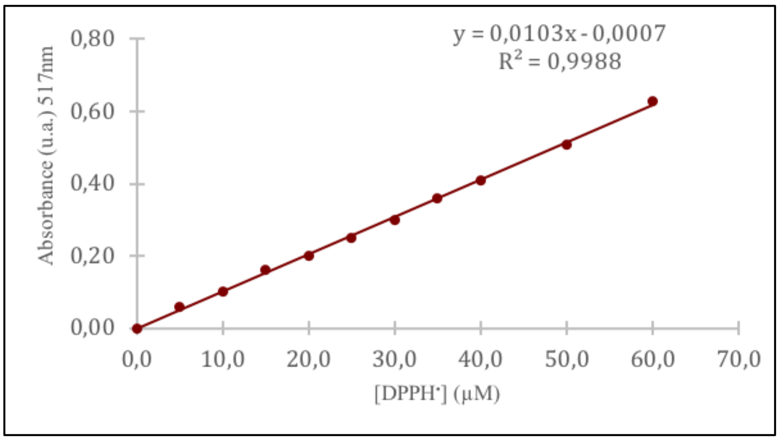

Figure SM-1 Calibration curve of DPPH• in methanol (MeOH).

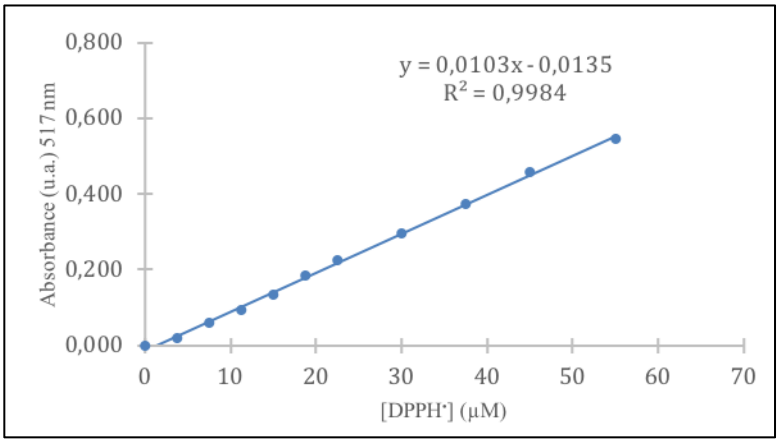

Figure SM-2 Calibration curve of DPPH• in acetonitrile (ACN).

3.2. DPPH assay in methanol

Once the linearity of the monitored method at 517 nm was evaluate by the calibration curve of DPPH• concentration versus absorbance at 517 nm, 2 mL of 140 μM DPPH• stock solution (70 μM DPPH final concentration) were added to the degassed methanol solutions (2mL) of antioxidant, with a final concentration from 0 to 60 μM. After 60 minutes stirring at room temperature in the absence of light, the absorbance was measured at 517 nm. Figure SM-3 shows the plot of inhibition percentage versus $mols_{antioxidant}/mols_{DPPH^\bullet}$ for the synthesized compound **9-11** and **15-20**.

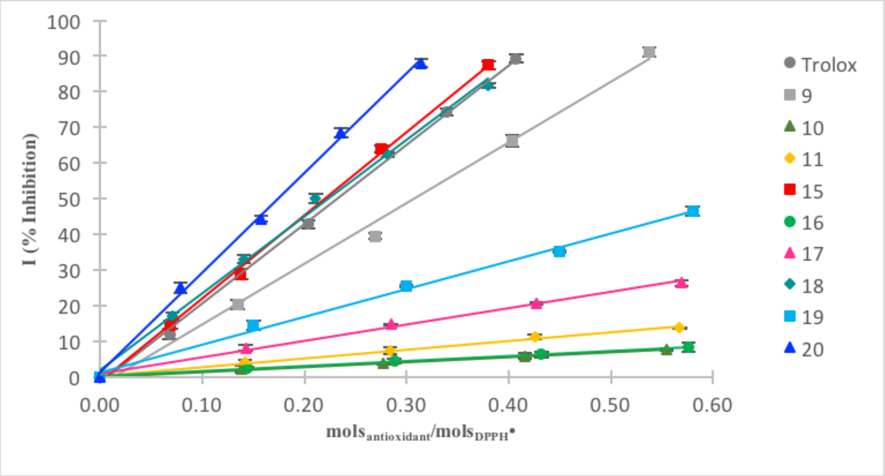

Figure SM-3 Regression line of the tested compounds **9-11** and **15-20** in DPPH assay in methanol.

As it is observed for Trolox, only compounds **9**, **15**, **18** and **20** achieve a ratio molar ($mols_{antioxidant}/mols_{DPPH^•}$) around 0.5, close to 100% of DPPH• inhibition, whereas the other exhibit poor reactivity towards the DPPH•. The estimated linear regression lines from curves plotted in Figure SM-3 and their coefficient of determination R^2, are shown in Table SM-1.

Table SM-1 Estimated regression lines from curves plotted in Figure SM-1.

Antioxidants	Regression line	R^2
**Trolox**	$\hat{y} = 222.53x - 1.5833$	0.999
**9**	$\hat{y} = 169.92x - 2.2668$	0.995
**10**	$\hat{y} = 13.79x + 0.0812$	0.998
**11**	$\hat{y} = 24.39x + 0.3898$	0.994
**15**	$\hat{y} = 233.18x + 1.2140$	0.998
**16**	$\hat{y} = 14.285x - 0.2239$	0.997
**17**	$\hat{y} = 45.914x + 0.8974$	0.995
**18**	$\hat{y} = 215.07x + 1.8629$	0.996
**19**	$\hat{y} = 77.934x + 1.3057$	0.995
**20**	$\hat{y} = 279.24x + 1.2797$	0.998

From the regression lines, the rIC_{50} in terms of $mols_{antioxidant}/mols_{DPPH^•}$ was estimated. Additionally, the ARP, the stoichiometry and the number of DPPH• reduced also were calculated as reported in Table SM-2.

Table SM-2 Antioxidant capacity of compounds **9-11** and **15-20** towards DPPH• in methanol.[a]

Antioxidant	rIC_{50} ($mols_{antioxidant}/mols_{DPPH•}$)	ARP	Stoichiometric value[b]	Number DPPH• reduced[c]
Trolox	0.23	4.31	0.46	2.16
**9**	0.31	3.25	0.62	1.63
**10**	3.62	0.28	7.24	0.14
**11**	2.03	0.49	4.07	0.25
**15**	0.22	4.55	0.44	2.28
**16**	3.52	0.28	7.03	0.14
**17**	1.07	0.94	2.14	0.47
**18**	0.24	4.15	0.48	2.07
**19**	0.62	1.60	1.25	0.80
**20**	0.18	5.45	0.37	2.72

[a]All the measures were performed in triplicate and the values were reported as mean ± SD. [b]The stoichiometry was derived by multiplying the rIC_{50} by two, to give the quantity of antioxidant that is need to reduce 100% of the DPPH•. [c]The number of DPPH• molecules, reduced by one molecule of antioxidant, is the inverse of the stoichiometry.

3.3. DPPH assay in acetonitrile

The assay was performed as described above, using as solvent the acetonitrile (ACN). Once the linearity of the monitored method at 517 nm was evaluate in this solvent through the calibration curve of DPPH• concentration *versus* absorbance at 517 nm, the absorbance measurements of each solution, containing a known concentration of antioxidant and DPPH•, were collected. Figure SM-4 displayed the plot of inhibition percentage *versus* $mols_{antioxidant}/mols_{DPPH}$• for the synthesized compounds **9-11** and **15-18**.

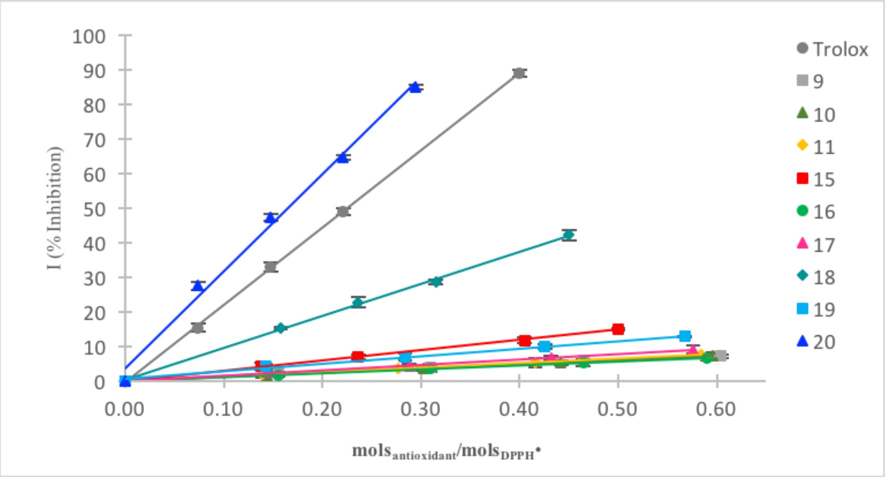

Figure SM-4 Regression line of the tested compound **9-11** and **15-20** in DPPH assay in acetonitrile.

Sequentially Table SM-3 and Table SM-4 show the corresponding regression lines with the values of R^2 as well as all the deducible parameters from rIC_{50}.

Table SM-3 Estimated regression lines from curves plotted in Figure SM-4.

Antioxidants	Regression line	R^2
**Trolox**	ŷ = 222.94x – 0.3162	0.999
**9**	ŷ = 11.677x + 0,2801	0.993
**10**	ŷ = 12.122x - 0.064	0.996
**11**	ŷ = 12,732x + 0.0495	0.997
**15**	ŷ = 29,592x + 0,0397	0.999
**16**	ŷ = 11.189x – 0.0091	0.994
**17**	ŷ = 15.396x + 0.0339	0.995
**18**	ŷ = 92.456x + 0.286	0.998
**19**	ŷ = 22.203x + 0.4898	0.993
**20**	ŷ = 280.63x + 3.4775	0.992

From the regression lines, the rIC_{50} in terms of $mols_{antioxidant}/mols_{DPPH^\bullet}$ was estimated. Additionally, the ARP, the stoichiometry and the number of DPPH• reduced also were calculated as reported in Table SM-4.

Table SM-4 Antioxidant capacity of compounds **9-11** and **15-20** towards DPPH• in acetonitrile.[a]

Antioxidant	rIC_{50} ($mols_{antioxidant}/mols_{DPPH^\bullet}$)	ARP	Stoichiometric value[b]	Number DPPH• reduced[c]
Trolox	0.22	4.49	0.45	2.24
**9**	4.26	0.23	8.52	0.12
**10**	4.12	0.24	8.25	0.12
**11**	3.92	0.25	7.85	0.13
**15**	1.69	0.59	3.38	0.30
**16**	4.47	0.22	8.94	0.11
**17**	3.25	0.31	6.49	0.15
**18**	0.54	1.86	1.08	0.93
**19**	2.23	0.45	4.46	0.22
**20**	0.17	6.03	0.33	3.02

[a]All the measures were performed in triplicate and the values were reported as mean ± SD. [b]The stoichiometry was derived by multiplying the rIC_{50} by two, to give the quantity of antioxidant that is need to reduce 100% of the DPPH•. [c]The number of DPPH• molecules, reduced by one molecule of antioxidant, is the inverse of the stoichiometry

3.4. Measurements of Rate Constants for the reaction of compounds ***9*****,** ***15*****,** ***18*****,** *and* ***20*** *with DPPH•*

The procedure used to determine k^s was common to all solvents and compounds. A solution of DPPH• and the compound were prepared in nitrogen-purged solvents. Then, the decay of DPPH• in the presence of a known concentration of compound was followed at 517 nm on a Shimadzu-UV-2401PC spectrophotometer. The concentration of DPPH• was $8.5*10^{-5}$ M and the compounds were always used in large excess over [DPPH•]. The decay of the DPPH• absorbance was analysed as pseudo-first-order processes to yield k_{ex}/s^{-1}, using *Sigma plot* for Windows. The measurements were performed in triplicate. Plots of k_{ex} *vs* compound concentration is linear and its slopes gave the second-order rate constants, k^s. The observed results are reported in Table SM-5.

Table SM-5 Kinetics data for the reaction of DPPH• with compounds **9**, **15**, **18**, and **20** in methanol.

Compounds	Conc. (mM)	k_{ex}[a]	k^s ($M^{-1}s^{-1}$)	R^{2b}
9	1.5	18.14 ± 0.7	3.26	0.9952
	2	20.40 ± 0.6		
	3	23.70 ± 0.9		
	4.5	27.67 ± 0.6		
	6	33.50 ± 9.5		
15	1	6.06 ± 0.2	3.77	0.9980
	1.5	6.20 ± 0.4		
	2	6.43 ± 0.2		
	3	6.78 ± 0.5		
	4	7.18 ± 0.1		
18	1.5	5.21 ± 0.01	1.40	0.9945
	2	6.07 ± 0.1		
	3	7.17 ± 0.3		
	4	8.63 ± 0.5		
	5	10.27 ± 0.6		
20	1	7.06 ± 0.02	0.77	0.9968
	1.22	7.28 ± 0.04		
	1.5	7.60 ± 0.02		
	2.5	8.30 ± 0.03		
	4	9.40 ± 0.05		

[a] First order rate constant $k_{ex} = 10^3\ k_{experimental}$/ s^{-1}; [b] Regression coefficient for each data set.

Table SM-6 Kinetics data for the reaction of DPPH• with compounds **9**, **15**, **18**, and **20** in acetonitrile.

Compounds	Conc. (mM)	k_{ex}[a]	k^s ($M^{-1}s^{-1}$)	$R^{2[b]}$
**9**	1	6.19 ± 0.2	8.54	0.9906
	1.5	11.30 ± 0.3		
	2	16.98 ± 0.3		
	3	25.05 ± 0.3		
	4	31.90 ± 0.2		
**15**	1.7	2.23 ± 0.2	1.26	0.9986
	2	2.57 ± 0.2		
	3	4.03 ± 0.2		
	4	5.25 ± 0.3		
	6	7.66 ± 0.2		
**18**	1	2.72 ± 0.06	0.23	0.9962
	2	3.00 ± 0.07		
	3	3.31 ± 0.06		
	4	3.49 ± 0.04		
	6	4.07 ± 0.04		
**20**	1	7.58 ± 0.12	2.22	0.9935
	2	10.29 ± 0.59		
	3	12.23 ± 0.26		
	4	14.24 ± 0.43		
	6	18.75 ± 0.05		

[a] First order rate constant $k_{ex} = 10^3\ k_{experimental}$/ s^{-1}; [b] Regression coefficient for each data set.

References

1. Vetica, F.; Pelosi, A.; Gambacorta, A.; Loreto, M.A.; Miceli, M.; Gasperi, T. Catalytic friedel-crafts/lactonization domino reaction: Facile access to 3-hydroxybenzofuran-2-one scaffold. *Eur. J. Org. Chem.* **2014**, *2014*, 1899-1906.
2. Vetica, F.; de Figueiredo, R.M.; Cupioli, E.; Gambacorta, A.; Loreto, M.A.; Miceli, M.; Gasperi, T. First asymmetric organocatalyzed domino friedel-crafts/lactonization reaction in the enantioselective synthesis of the GABA(b) receptor modulator (s)-BHFF. *Tet. Lett.* **2016**, *57*, 750-753.
3. Dyachenko, V.I.; Kolomiets, A.F.; Fokin, A.V. Preparation of substituted 3-hydroxy-3-trifluoromethyl-2(3H)benzofuranones. *Bull. Acad. Sc. Ussr Div. Chem. Sc.***1987**, *36*, 2332-2337.
4. Dyachenko, V.I.; Galakhov, M.V.; Kolomiets, A.F.; Fokin, A.V. 2-hydroxy-2-trifluoromethyl-2,3h-1,4-benzoxazinone-3. *Bull. Acad. of Sc. Ussr Div. Chem. Sc.* **1988**, *37*, 1056-1056.
